# 

**Published:** 1892-11-26

**Authors:** 


					'^itroU at the General Post Office a? a Newspaper tut j??>
tt^' nrniimiiin m Iho Uiutoil Kin^d^m axui Abroad.J
n tAIRA INUROIIHU OUrrLCMLi
Per Annum, 83. 6d., post fiee.
Monthly Parts, 6d.; post free, 8d.
Ditto per Annum, 8s , post free.
No. 322. Vol. XIII.] Sattjruay,
November 26th, 1892.
("Twopence.
...

				

